# Next-generation sequencing-based evaluation of the actionable landscape of genomic alterations in solid tumors: the “MOZART” prospective observational study

**DOI:** 10.1093/oncolo/oyae206

**Published:** 2024-08-23

**Authors:** Francesco Schettini, Marianna Sirico, Marco Loddo, Gareth H Williams, Keeda-Marie Hardisty, Paul Scorer, Robert Thatcher, Pablo Rivera, Manuela Milani, Carla Strina, Giuseppina Ferrero, Marco Ungari, Cristina Bottin, Fabrizio Zanconati, Nicolò de Manzini, Sergio Aguggini, Richard Tancredi, Elena Fiorio, Antonio Fioravanti, Maurizio Scaltriti, Daniele Generali

**Affiliations:** Translational Genomics and Targeted Therapies in Solid Tumors Group, August Pi i Sunyer Biomedical Research Institute (IDIBAPS), 08036 Barcelona, Spain; Medical Oncology Department, Hospital Clinic of Barcelona, 08036 Barcelona, Spain; Faculty of Medicine and Health Sciences, University of Barcelona, 08036 Barcelona, Spain; Department of Medical Oncology, IRCCS Istituto Romagnolo per lo Studio dei Tumori (IRST) “Dino Amadori,” 47014, Meldola, Italy; Oncologica UK Ltd, Cambridge CB10 1XL, United Kingdom; Oncologica UK Ltd, Cambridge CB10 1XL, United Kingdom; Oncologica UK Ltd, Cambridge CB10 1XL, United Kingdom; Oncologica UK Ltd, Cambridge CB10 1XL, United Kingdom; Oncologica UK Ltd, Cambridge CB10 1XL, United Kingdom; Medical Oncology Department, Hospital Clinic of Barcelona, 08036 Barcelona, Spain; Department of Medical, Surgical and Health Sciences, University of Trieste, 34147, Trieste, Italy; Department of Medical, Surgical and Health Sciences, University of Trieste, 34147, Trieste, Italy; Multidisciplinary Unit of Breast Pathology and Translational Research, Cremona Hospital, 26100, Cremona, Italy; Multidisciplinary Unit of Breast Pathology and Translational Research, Cremona Hospital, 26100, Cremona, Italy; Department of Medical, Surgical and Health Sciences, University of Trieste, 34147, Trieste, Italy; Department of Medical, Surgical and Health Sciences, University of Trieste, 34147, Trieste, Italy; Department of Medical, Surgical and Health Sciences, University of Trieste, 34147, Trieste, Italy; Multidisciplinary Unit of Breast Pathology and Translational Research, Cremona Hospital, 26100, Cremona, Italy; Multidisciplinary Unit of Breast Pathology and Translational Research, Cremona Hospital, 26100, Cremona, Italy; Section of Oncology, Department of Medicine, University of Verona School of Medicine and Verona University Hospital Trust, 37134, Verona, Italy; Pathology Unit, ASST Cremona, 26100, Cremona, Italy; Neurosurgery Unit, ASST Cremona, 26100, Cremona, Italy; AstraZeneca, Gaithersburg, MD 20876, United States; Department of Medical, Surgical and Health Sciences, University of Trieste, 34147, Trieste, Italy; Multidisciplinary Unit of Breast Pathology and Translational Research, Cremona Hospital, 26100, Cremona, Italy

**Keywords:** solid tumors, next-generation sequencing, ESCAT, clinical actionability, molecular profiling, metastatic

## Abstract

**Background:**

The identification of the most appropriate targeted therapies for advanced cancers is challenging. We performed a molecular profiling of metastatic solid tumors utilizing a comprehensive next-generation sequencing (NGS) assay to determine genomic alterations’ type, frequency, actionability, and potential correlations with PD-L1 expression.

**Methods:**

A total of 304 adult patients with heavily pretreated metastatic cancers treated between January 2019 and March 2021 were recruited. The CLIA-/UKAS-accredit Oncofocus assay targeting 505 genes was used on newly obtained or archived biopsies. Chi-square, Kruskal-Wallis, and Wilcoxon rank-sum tests were used where appropriate. Results were significant for *P* < .05.

**Results:**

A total of 237 tumors (78%) harbored potentially actionable genomic alterations. Tumors were positive for PD-L1 in 68.9% of cases. The median number of mutant genes/tumor was 2.0 (IQR: 1.0-3.0). Only 34.5% were actionable ESCAT Tier I-II with different prevalence according to cancer type. The DNA damage repair (14%), the PI3K/AKT/mTOR (14%), and the RAS/RAF/MAPK (12%) pathways were the most frequently altered. No association was found among PD-L1, ESCAT, age, sex, and tumor mutational status. Overall, 62 patients underwent targeted treatment, with 37.1% obtaining objective responses. The same molecular-driven treatment for different cancer types could be associated with opposite clinical outcomes.

**Conclusions:**

We highlight the clinical value of molecular profiling in metastatic solid tumors using comprehensive NGS-based panels to improve treatment algorithms in situations of uncertainty and facilitate clinical trial recruitment. However, interpreting genomic alterations in a tumor type-specific manner is critical.

## Teaser

The identification of the most appropriate targeted therapies for advanced cancers is challenging. The authors performed a molecular profiling of metastatic solid tumors utilizing a comprehensive next-generation sequencing (NGS) assay to determine genomic alterations’ type, frequency, actionability, and potential correlations with PD-L1 expression.

**Implications for practice**

We showed in pretreated metastatic solid tumors a potentially actionable mutation detection rate of 78%. However, clinically actionable ESCAT Tier I-II genomic alterations were detected in 36% cases, with a detection rate >10% only in melanoma, NSCLC, breast, and prostate cancer. No association was found with PD-L1 status. Only 20% of patients was treated according to molecular results, with limited objective responses. The same matched treatments in different cancers with similar molecular alterations resulted in heterogeneous responses. This study supports molecular profiling when scarce therapeutic alternatives are available, but points out the need for molecular tumor boards to optimize therapeutic decision-making.

## Introduction

When chemotherapy was first introduced for the treatment of solid tumors, the new and unexpected survival benefits observed prioritized the need to develop less toxic and more targeted therapeutic approaches.^1^ Subsequently, improved screening programs, novel systemic treatments, refinement of surgical, and radiotherapeutic techniques, along with more integrated multidisciplinary therapeutic approaches, have led to a visible increase in patients’ survival in the last 3 decades, despite geographical disparities and differences according to cancer type.^2^ This has led the scientific community to pursue the identification of increasingly personalized treatments to maximize therapeutic efficacy while sparing patients with unnecessary toxic treatments. In this perspective, a recent exponential increase in both tumor molecular profiling and discovery of effective molecularly targeted agents has provided new therapeutic opportunities for patients with advanced cancers harboring specific somatic and/or germline mutations.^3^ Examples are represented by PARP inhibitors in germline *BRCA*1/2-mutant ovarian, breast, prostate, or pancreatic cancer,^4^ the PI3K-inhibitor (PI3Ki) alpelisib for *PIK3CA*-mutant hormone receptor-positive (HR+)/HER2-negative breast cancer (BC), vemurafenib for *BRAF*-mutant melanoma,^5^ or the tyrosine kinase inhibitors entrectinib and larotrectinib, for solid tumors carrying NTRK fusions.^6^

The identification of the most appropriate targeted therapies is challenging as a consequence of the limited coverage of most molecular profiling tests used in routine clinical practice and the frequent lack of direct evidence-based linkage with therapy.^3^ These technical limitations translate into the loss of potential therapeutic opportunities for many patients. At our center, we have addressed this challenge by performing precision oncology genomic testing utilizing a comprehensive precision oncology next generation sequencing (NGS) assay,^7,8^ to identify the prevalence of potentially actionable genomic modifications in a large cohort of patients with heavily pretreated metastatic solid tumors, where therapeutic options are usually limited. Here we report the analysis of test trending data from this large screening program to investigate the prevalence of actionable molecular alterations directed at either the prescription of specific salvage targeted treatments (off-label or in indication) or alternatively to recruit patients to the most appropriate clinical trials. The NGS genomic data was also analyzed in combination with a PD-L1 companion diagnostic (CDx) immunohistochemistry (IHC) test, in order to assess potential correlations between clinically actionable genomic alterations and PD-L1 expression, a biomarker of response to anti-PD1/PD-L1 immune-checkpoint inhibitors (ICI).^9^

## Materials and methods

### Study design and objectives

This was a prospective observational study, named “the MOZART Trial,” that recruited adult patients with advanced solid tumors treated at the Oncology Unit of the Cremona Hospital (Italy) between January 2019 and March 2021. Patients had to be already pretreated with at least 2 standard-of-care treatment lines for their metastatic disease and present with at least 1 accessible metastatic lesion to perform a biopsy. If not, archived formalin-fixed paraffin-embedded (FFPE) tumor tissue from the latest biopsy received was used. More detailed inclusion/exclusion criteria are reported in Supplementary Methods. The NGS and PD-L1 assays were applied to the tumor biopsies as described below. The primary objective of the present observational study was to assess the prevalence of actionable molecular alterations in solid tumors in later treatment lines. Secondary objectives were to detect the levels of PD-L1 and potential association with specific actionable genomic modifications. To guide therapeutic decision-making was not the purpose of the present study. However, treating physicians were informed of the test results. If patients were deemed to be sufficiently fit to receive a further treatment line, physicians could then ask permission from their institution for off-label prescription or compassionate drug use, unless an on-label matched mutation treatment was available. If possible, patients were recruited in clinical trials, as well. PD-L1 assessment for immunotherapy prescription was repeated with an approved PD-L1 CDx assay whenever required, according to tumor type, treatment line, and regulatory approval in Italy. An exploratory description of the clinical outcomes for a subgroup of patients receiving matched treatments has been here provided. However, a detailed report of matched treatments-genomic alterations and clinical outcomes was beyond the scope of this study, hence data were limited. Nevertheless, we reported with more detail the experience of 2 patients who provided their informed consent to publish their anonymized clinical data, as an example of NGS testing advantages and pitfalls.

### Comprehensive NGS genomic profiling

NGS genomic profiling including DNA extraction, library preparation, sequencing, and data analysis was centrally performed on FFPE tumor tissue. The Oncofocus assay (Oncologica UK Ltd, Chesterford Research Park, Cambridge, UK), specifically adapted for the present molecular screening, is validated for clinical use and accredited by CLIA (ID 99D2170813) and by UKAS (9376) in compliance with ISO15189:2012 and following the guidelines published by the Association for Molecular Pathology and College of American Pathologists and IQN-Path ASBL.^10,11^ It is a NGS-based platform designed to detect druggable genomic alterations, according to Food and Drug Administration and European Medicine Agency approvals, European Society for Medical Oncology (ESMO), and National Comprehensive Cancer Network guideline references or ongoing clinical trials worldwide and is updated every 12 weeks.^7,8^ It targets 505 genes and detects actionable genetic variants linked to 700 anti-cancer targeted therapies or combinations. For the purpose of this study, all genomic alterations detected were manually classified by using the ESMO Scale for Clinical Actionability of Molecular Targets (ESCAT), according to the latest guidelines and evidence found^12^ (Supplementary references). DNA and RNA extraction, library preparation and sequencing, quality control metrics, data analysis methods, and the list of genes and genomic alterations covered are reported as Supplementary methods.

### Immunohistochemistry for PD-L1 expression and tumor mutational burden assessment

The assessment of PD-L1 immunostaining was performed by a qualified histopathologist at Oncologica (G.W.), in accordance with PD-L1 clinical reporting guidelines,^13^ to quantify the proportion of tumor cells expressing PD-L1 (tumor proportion score) and the area occupied by tumor-infiltrating PD-L1 positive immune cells in FFPE samples. According to the tumor proportional score (TPS), all cases were subdivided into PD-L1 negative (TPS < 1%), positive-low (TPS ≤ 1%≤TPS ≤ 49%) and positive-high (TPS ≥ 50%).^14,15^ The rabbit monoclonal antibody clone E1L3N used for PD-L1 assessment is not licensed and approved for use in clinical testing to direct the use of PD1/PD-L1 therapies (Supplementary methods).

Tumor mutational burden (TMB) is usually defined as the number of somatic mutations per megabase (Mb) of interrogated genomic sequence and is a potential biomarker of response to ICI.^9^ The Oncofocus assay covers ~1.2 Mb over 505 genes, complying with the evidence-based suggestion that gene panels of at least 1 Mb are needed for TMB measurement.^16-18^ Ten mutations/Mb was the cutoff to define high vs low TMB cases.

### Statistical analysis

Descriptive statistics, Chi-square, Kruskal-Wallis, or Wilcoxon rank-sum test with continuity correction were used where appropriate. Univariate logistic regressions were performed to assess associations between mutational or PD-L1 status and variables of interest. Statistical significance was set at *P* < .05. Progression-free interval (PFI) was defined as the time from initiation of matched treatment to the occurrence of disease progression or death.^19^ For all analyses Microsoft Excel 2019 vers.16.50 and R vers.3.6.1 for MacOS X were used.

## Results

### Population characteristics

Overall, 304 patients were included in this study. Median age was 56.3 years (interquartile range [IQR]: 59.0-68.4) and patients were predominantly female (59.5%). All patients were sufficiently fit (ECOG 0-2) to be treated with anticancer agents and were candidates to receive a salvage therapy or best supportive care in the absence of suitable late-line treatments.

A total of 237 tumors (78%) harbored potentially actionable genomic modifications detected with the NGS assay platform. BC was the most common tumor type, representing 25.3% of the entire cohort, followed by colorectal cancer (CRC) (12.5%), ovarian cancer (7.9%), prostate cancer (6.9%), primary malignancies of the central nervous system (6.6%), and non-small cell lung cancer (NSCLC) (5.3%). With a prevalence <5% but >2%, there were patients affected by bladder, pancreatic, kidney, biliary tract, cervical/vulvovaginal cancer, head and neck (H&N) tumors, soft-tissues sarcomas from several origins (abdominal, H&N, lung), and gastric cancer. Tumors with a prevalence ≤2% in our cohort were small cell lung cancer (SCLC), endometrial, liver, non-melanoma skin cancers, melanoma, mesothelioma, cancer of unknown primary, neuroendocrine tumors (NET) from distinct origins (pancreas, lung, intestine, unknown), thymic carcinoma, and penile cancer. The main population characteristics are detailed in Table 1.

**Table 1.** Patients characteristics.

Characteristics	Overall population	
	N	%
	304	100.0
Age		
Median	56.3	—
IQR	59.0-68.4	—
**Sex**		
Female	181	59.5
Male	123	40.5
Cancer type*		
Carcinomas	268	88.2
Non-carcinomas (excluding hematologic malignancies)	36	11.8
Treatment line		
Median	3	—
Min-max	3-5	—
Mutational status		
Mutant	237	78.0
Wild-type	67	22.0
Genomic alteration per tumor		
Median	2	—
IQR	1-3	—
Range	0-8	—
PD-L1 status		
Median %	1.0	—
IQR	0.0-3.0	—
Min-max	0.0-100.0	—
Negative (TPS < 1%)	87	31.1
Positive-low (1%≤TPS < 49%)	173	61.8
Positive-high(TPS ≥ 50%)	20	7.1
Overall	280	92.1

*Cancer types and frequency are detailed in Table 2.

Abbreviations: CNS, central nervous system; CUP, cancer of unknown primary; IQR, interquartile range; max, maximum; min, minimum; NSCLC, non-small cell lung cancer; SCLC, small cell lung cancer; TPS, tumor proportion score; TPS, tumor proportion score

**Figure 1.**
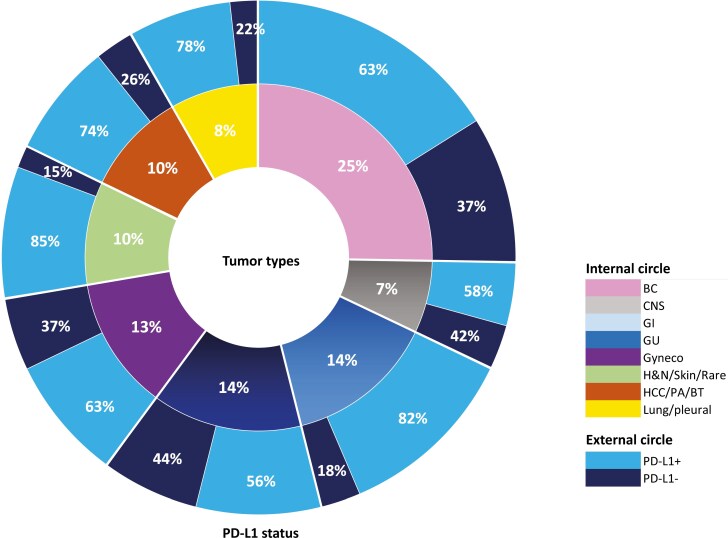
PD-L1 status according to tumor groups. External circle’s % are referred to the proportion of PD-L1+ and —patients within each tumor type/group. Internal circle’s % are referred to the frequency of a specific tumor type/group with respect to the overall patients cohort. Abbreviations: +, positive; −, negative; BC, breast cancer; CNS, central nervous system primary tumors; GI, gastrointestinal; GU, genitourinary (including prostate); Gyneco, gynecological malignancies; H&N, head and neck; HCC, hepatocellular carcinoma; PA, pancreatic adenocarcinoma; BT, biliary tract; skin, includes melanoma and non-melanoma skin cancers.

Tumors were positive for PD-L1 according to TPS in 68.9% cases, with high levels (TPS ≥ 50%) observed in 7.1%. In 24 cases, the material to analyze both PD-L1 and tumor mutational status was insufficient. In these cases, the NGS assay was prioritized. The distribution of PD-L1-positive (+) vs PD-L1-negative (−) tumors across cancer types did not reach statistical significance (*P* = .065) (Figure 1). Still, 2 macro groups with significant difference in PD-L1 status could be ultimately identified (*P* < .001). Namely, a group including BC, CNS, genitourinary/prostate (GU),and gynecological malignancies (80% PD-L1+) and a group including gastrointestinal (GI), liver, pancreatic, biliary tract, pulmonary, pleural, H&N, skin, and rare tumors (61% PD-L1+).

### Molecular results

Overall, 236 tumors harbored at least 1 molecular alteration involving genes screened by the Oncofocus panel (Table 2).

**Table 2.** Detailed cancer type incidence in the study cohort.

Characteristics	Overall population		Mutant population	
	*N*	%*	*N*	%#
	304	100.0	236	77.6
Cancer type				
Breast	77	25.3	62	80.5
Colorectal, anal, and small intestine	38	12.5	36	94.7
Ovarian	24	7.9	16	66.7
Prostate	21	6.9	7	33.3
CNS primary	20	6.6	18	90.0
NSCLC	16	5.3	14	87.5
Bladder/ureter	9	4.9	8	88.9
Pancreas	15	3.3	13	86.7
Kidney	10	3.0	7	70.0
Gallbladder and biliary tract	9	3.0	6	66.7
Cervix/vagina	7	3.0	4	57.1
Sarcoma	9	2.3	6	66.7
Head and neck	7	2.3	5	71.4
Gastric	7	2.3	6	85.7
SCLC	5	2.0	5	100.0
Liver	6	1.6	4	66.7
Mesothelioma	4	1.6	3	75.0
Uterine	5	1.3	5	100.0
Melanoma	3	1.3	3	100.0
Skin non-melanoma	2	1.3	1	50.0
CUP	4	1.0	2	50.0
Neuroendocrine	4	0.7	4	100.0
Thymic	1	0.3	1	100.0
Penis	1	0.3	0	0.0

*The proportions in the overall study cohort are referred to the frequency of each specific cancer type with respect to the total population of 304 patients.

#The proportions in the mutant study cohort are referred to the total of each cancer type.

Abbreviations: CNS, central nervous system; CUP: cancer of unknown primary; NSCLC, non-small cell lung cancer; SCLC, small cell lung cancer.

A total of 549 genomic alterations were detected (full list available as Supplementary Table S1), along with 1 case of high TMB (TMB-H) in a NSCLC. In multiple cases the same mutation was detected in different tumors, leading to a total of 272 distinct molecular alterations. The median number of altered genes *per* tumor was of 2.0 (IQR: 1.0-3.0, minimum-maximum range [min-max]: 0.0-8.0). The median mutational frequency rate was 77.8% (IQR: 66.7%-92.1%; min-max: 0.0%-100.0%). The tumors in the lower quartile (Q1) were ovarian, gallbladder/biliary tract, liver, cervix/vaginal, non-melanoma skin cancers, sarcomas, CUPs, penis, and prostate cancer. In the following quartile (Q2) were included mesothelioma, kidney, and H&N cancers, while in the subsequent quartile (Q3) BC, NSCLC, CNS primary tumors, bladder/ureter, pancreatic, and gastric cancer were included. The upper quartile (Q4) included colorectal/anal/small intestine, uterine, thymic, neuroendocrine cancers, SCLC, and melanomas. As expectable, the difference in mutant vs non-mutant tumors for each quartile group was significantly higher as the quartile increased (*P* < .001), with an increasing proportion *per* quartile of cases harboring at least one genomic alteration in covered genes (Table 3).

**Table 3.** Proportion of mutant tumors *per* mutational frequency quartiles and distribution of actionable molecular alterations *per* quartile.

Mutational status	Q1		Q2		Q3		Q4		*P*
	*N*	%	*N*	%	*N*	%	*N*	%	
Mutant tumors	46	55.4	15	71.4	121	84.0	54	96.4	<.001
Non-mutant tumors	37	44.6	6	28.6	23	16.0	2	3.6	
Total number of patients	83	27.3	21	6.9	144	47.4	56	18.4	
ESCAT I-II molecular alterations	6	5.9	1	3.2	69	30.1	5	4.2	<.001
ESCAT ≥ III molecular alterations	96	94.1	30	96.8	160	69.9	113	95.8	
Total number of molecular alterations	102	100.0	31	100.0	229	100.0	118	100.0	

Q, quartile; Q1: ≤66.7% mutant tumors *per* cancer type; Q2: >66.7% and ≤77.8% mutant tumors *per* cancer type; Q3: >77.8% and ≤91.2% mutant tumors *per* cancer type; Q4: >91.2% and ≤100.0% mutant tumors *per* cancer type.

Consistently, a statistically significant difference in the median number of genomic alterations according to tumor type was observed (*P* < .001), with CNS tumors showing a median of 3.0 (IQR: 1.75-3.25), followed by GI tumors presenting with a median of 2.0 (IQR: 2.0-3.0), breast tumors showing a median of 2.0 altered genes *per* tumor (IQR: 1.0-3.0), liver, pancreatic, and biliary tract cancers exhibiting a median of 2.0 (IQR: 1.0-2.0). The number of different genomic alterations according to sex and age (*P* = .279 and *P* = 0.098, respectively) did not differ significantly.

**Figure 2.**
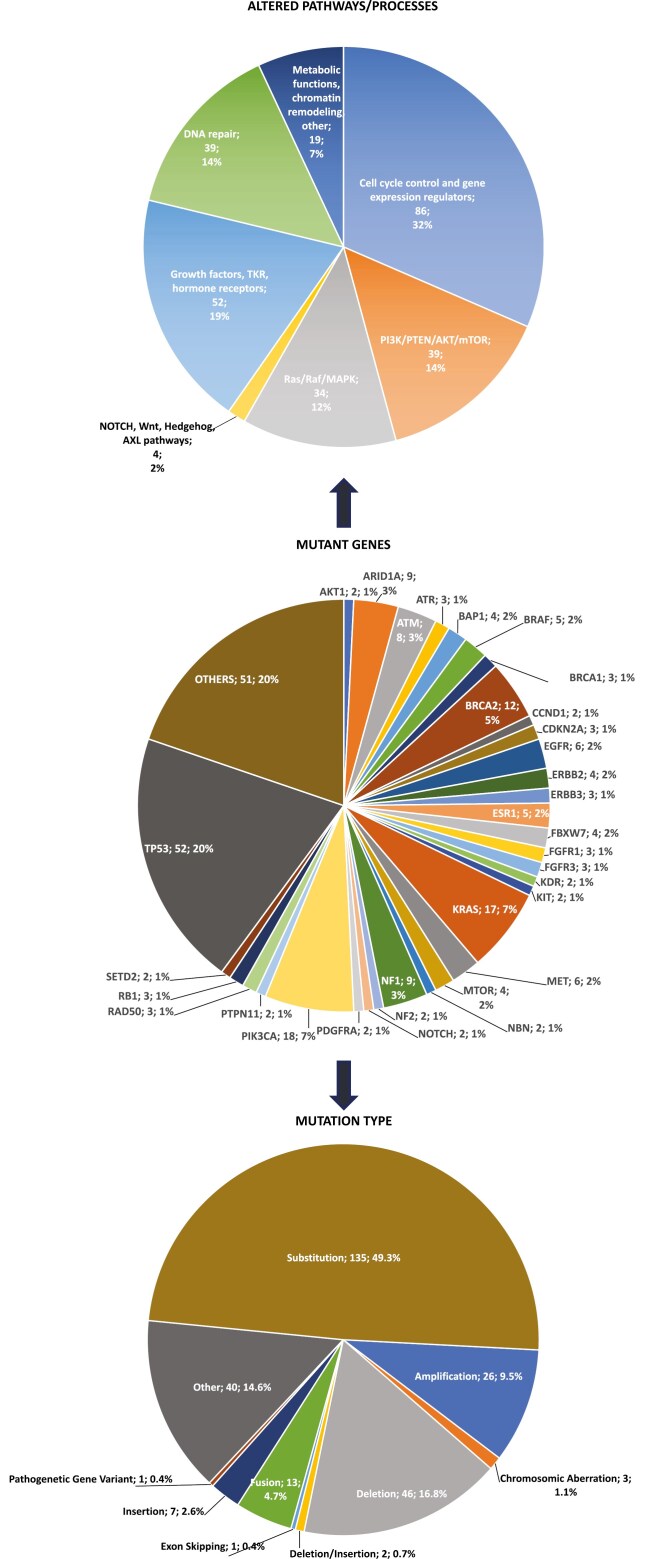
Mutational landscape. In the central pie plot, OTHERS includes all mutant genes with a prevalence <0.8% and values are rounded. Abbreviation: TKR, tyrosine kinase receptor.

The pathways most frequently involved were related to DNA damage repair (14%), the PI3K/AKT/mTOR (14%), and the RAS/RAF/MAPK (12%) pathways. Moreover, many genes involved in cell cycle control and gene expression regulation were found mutated (32% cases). In fact, the most frequently altered gene was *TP53,* mutated in 20.2% patients, followed by *BRCA2* (7.2%), *PIK3CA* (7%), *KRAS (*6.6%), *ARID1A* (3.5%), *NF1* (3.5%), and *ATM* (3.1%) (Figure 2). Alterations in cancer-related genes were derived from a range of different mechanisms, including substitution, deletion, amplification, fusion, insertion, chromosomic aberration, deletion/insertion, exon skipping, and pathogenetic gene variants. Substitutions were the predominant molecular alterations, detected in 49.3% of the samples, followed by deletions (16.8%), amplifications (9.5%), gene fusions (4.7%), insertions (2.6%), chromosomic aberrations (1.1%), and other types of mutations with lower frequency but collectively representing 14.6% of cases (Figure 2).

Regarding PD-L1, we did not detect any association between PD-L1 status (positive vs. negative) and tumor mutational status (*P* = 0.454) or a difference in genomic modification number according to PD-L1 category (*P* = 0.717). PD-L1 TPS score as a continuous variable was also not associated with mutational status (*P* = 0.840).

### Actionability of the detected genomic alterations

**Figure 3.**
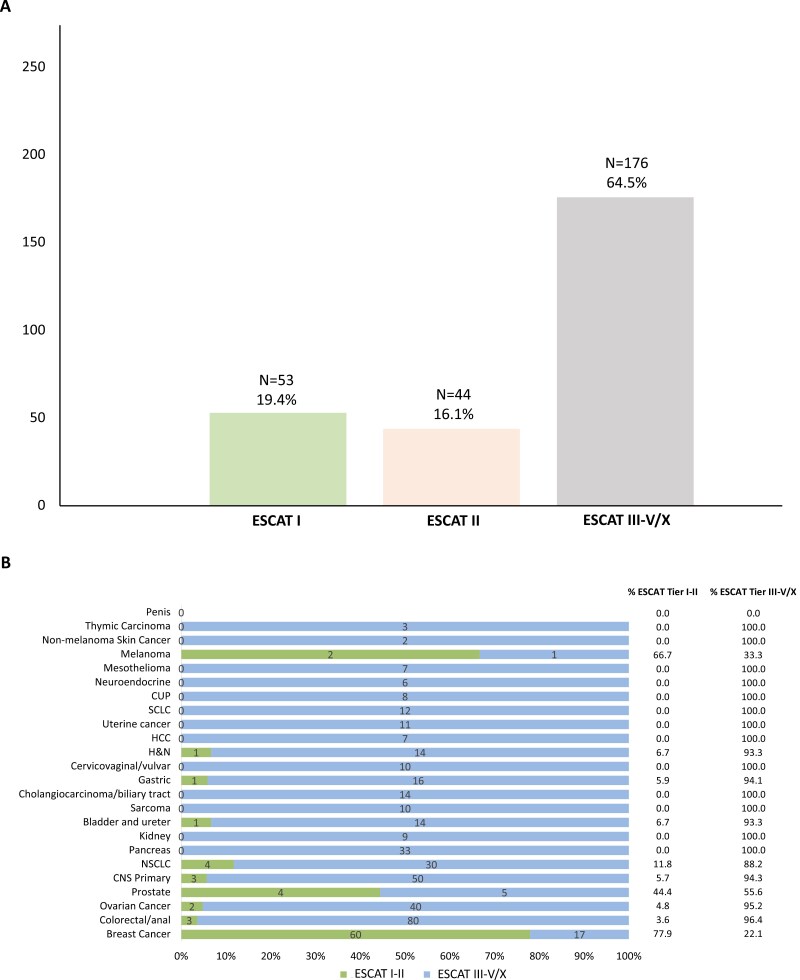
Actionability of detected mutations according to the ESCAT scale. A: Global proportion of different ESCAT tiers in the mutant cohort. Here, the best ESCAT tier for each single mutation detected was considered, independently of the tumor where it was detected. B: Prevalence of ESCAT I-II and III-V or X mutations according to tumor. Abbreviations: ESMO, European Society for Medical Oncology; ESCAT, ESMO Scale for Clinical Actionability of Molecular Targets; CNS, central nervous system primary tumors; H&N, head and neck; HCC, hepatocellular carcinoma; NSCLC, non-small cell lung cancer; SCLC, small cell lung cancer; CUP, cancer of unknown primary.

When considering the ESCAT scale to evaluate the clinical actionability of our cohort’s mutational landscape, we observed that among the total 273 different alterations identified (including TMB-H), only 19.4% were considerable as Tier I for at least one cancer type, while 16.1% were ESCAT Tier II, 6.6% ESCAT Tier III, and the rest was Tier IV-V or X (Figure 3). When we matched the molecular alteration with the respective ESCAT Tier according to the tumor where the alteration was actually detected, we observed that mutant non-breast tumors were significantly less likely to be associated with ESCAT Tier I-II molecular targets (*P* < .001). In fact, actionable ESCAT Tier I-II alterations in relevant proportion (>10%) were detected only for BC (77.9%), melanoma (66.7%), prostate cancer (44.4%), and NSCLC (11.8%). ESCAT Tier I-II alterations were found also in H&N (6.7%), bladder (6.7%), gastric (5.9%), CNS (5.7%) ovarian (4.8%), and CRC (3.6%) in <7% of cases. In the remaining tumor types, only molecular alterations of ESCAT Tier III-V or X were found (Figure 3). Consistently, when subdividing cancer types according to mutational frequency, a higher proportion of genomic modifications was not necessarily a synonym of actionability, since the vast majority of actionable ESCAT-I/II molecular alterations were found in the Q3 group (30.1%; *P* < .001) rather than Q4. In fact, the proportion of actionable alterations *per* group was low and similar among Q1, Q2, and Q4 (5.9, 3.2, 4.2%, respectively) (Table 3). The Q3 group included BC, NSCLC, CNS primary tumors, bladder/ureter, pancreatic, and gastric cancer; the higher actionability was driven by BC and NSCLC (Figure 3). Finally, no association was found between PD-L1 status and the presence of ESCAT tier I-II molecular alterations (*P* = .148).

### Examples of matched treatments

Overall, 62 out of 304 (20.4%) patients were treated according to the genomic assay or PD-L1 test result through an on-label, off-label, or compassionate use prescribing procedure, or in the context of a clinical trial. When required, PD-L1 positivity was confirmed with an approved CDx. Of note, the positivity concordance was of 89%. Twenty-three (37.1%) patients obtained the most relevant clinical benefit, achieving partial or complete responses as their best response according to clinical judgment, with an mPFI of 32.0 months (95% CI, 18.5 months-84.0 months). These patients were affected by either BC, endometrial or parotid cancer, mesothelioma or glioblastoma. In these tumors, the test identified from 1 to 2 genomic alterations or PD-L1 positivity. The other 39 tested cancers (bladder, gallbladder, lung, prostate, H&N, ovary, melanoma, and sarcoma) obtained either disease progression or stability as their best response, with a mPFI of 16.0 months (95% CI, 6.0 months-23.0 months) for patients with stable disease. In these tumors, the test detected a PD-L1 positivity and/or several molecular modifications, suggesting the presence of different “passenger” genomic alterations, likely not directly involved into cancer progression and responsiveness to specific target therapies. Due to study protocol and related ethical constraints, we could not report further details on clinical practice therapeutic decision-making and patients outcomes with matched treatments. Nevertheless, 2 patients with different tumors harboring the same mutation and both treated with off-label abemaciclib gave consent to report their anonymized clinical outcomes. A first case, was a chemotherapy-refractory, *CDKN2B*-deleted metastatic parotid tumors, that received abemaciclib as fourth-line therapy. In this setting, the inhibition resulted in a PFI of 18.5 months and improved quality of life due to tumor shrinkage and symptomatic relief. The second case was a chemotherapy-refractory, *CDKN2B*-deleted metastatic endometrial adenocarcinoma treated with abemaciclib as third-line therapy. In this setting, the inhibition resulted in a poor PFI of 2.5 months, with no substantial improvement in quality of life.

## Discussion

Our MOZART prospective observational study performed at the Cremona Hospital has demonstrated that precision oncology molecular profiling in advanced solid malignancies is of potential clinical value. The overall genomic alteration detection rate was 78%, with clinically actionable molecular alterations of ESCAT Tier I-II detected in 35.5% of cases with an additional 6.6% falling within the ESCAT Tier III category. BC, prostate cancer, melanoma, and NSCLC were the most frequently mutated tumors with clinically actionable alterations of ESCAT Tier I-II, followed by H&N, bladder, gastric, CNS, ovarian, and CRC. No associations were observed among PD-L1 status, age, sex, and tumor mutational status. GI and biliary tract tumors were the cancer types most frequently associated with PD-L1 positivity.

The genetic variants described in our study belong to some of the most common key cancer regulatory networks in solid tumors, such as the RAS/RAF/MAPK and PI3K/AKT/mTOR signaling pathways, the DNA damage repair (DDR) pathways, and cell cycle checkpoints. Notably, many approved or experimental targeted agents are precisely directed against these targets, for example, the PI3K inhibitors in BC, mTOR inhibitors in breast and kidney cancer, AKT inhibitors in BC, BRAF, and MEK inhibitors in melanoma or PARP inhibitors in several solid tumors.^4,20-24^ However, these drugs are costly, present with several side effects and are often administered without identification of the altered pathway of interest (eg, everolimus or capivasertib). This relates to the fact that the regulatory agencies have granted approval for these targeted agents without requirement for an accompanying CDx. Taking into account that their correct positioning in the therapeutic algorithms frequently presents many uncertainties, a broader implementation of molecular testing would greatly improve the selection of patients for these targeted agents and establish more personalized therapeutic algorithms when such uncertainty exists.

The rate of patients with ESCAT levels I-III identified in this study was comparable with other published screening programs.^10,25-28^ The ESCAT framework was promoted by a group of experts who reached a consensus on the criteria to define the clinical actionability of molecular alterations in solid tumors culminating in the establishment of the ESMO-ESCAT scale.^12^ In this way, clinicians now have the opportunity to become familiar with the type of genomic variants reported by genomic tests and their relevance for treatment. This is especially important following the results from the SHIVA and the SAFIR01 trials which both showed no improvement in progression-free survival (PFS)^29^ and disappointing overall response rates of only 9%^30^ in pretreated patients with advanced solid tumors, when the drug-genomic alteration match was not guided by sufficient evidence of clinical activity or efficacy. In fact, in our cohort, only 20% of patients ultimately received a mutation-matched treatment, with less than half experiencing an objective response. In this perspective, it is critical to interpret genomic alterations in a tumor type-specific manner. The most relevant example of this kind is the *BRAF V600E* mutation, which is considered Tier I for melanoma, since effective B-Raf inhibitors like dabrafenib and vemurafenib are already approved in the clinic as standard of care treatments.^31,32^ Conversely, it is considered Tier III for CRC, due to the limited activity of vemurafenib in this particular tumor type,^33^ and Tier II in cholangiocarcinoma, following positive results for the combination of dabrafenib and trametinib in the ROAR trial.^34^ An exception is represented by specific rare driver genomic alterations that can be targeted by molecular inhibitors approved in tumor-agnostic fashion, like *NTRK* fusions,^35,36^*RET* fusions^37^ and alterations in genes involved in the DNA mismatch repair mechanism.^38^

Importantly, a pooled analysis from the SAFIR02-BREAST and SAFIR-PI3K trials showed a significant PFS benefit (60% reduction in the risk of progression and death, *P* < .001) only in the presence of ESCAT Tier I-II alterations.^39,40^ These results highlight the importance of avoiding an excess of off-label prescription of target drugs, especially if only preclinical evidence of activity exists or only activity/efficacy in other cancer types has been proved. At the same time, targeted treatments with a good preclinical rationale should not be completely discarded in cases with very limited therapeutic options. The 2 clinical experiences reported within our genomic profiling program support both the concept that genomic testing for off-label drug prescription should be adopted with caution, as well as the concept that such a genomic profiling program might represent an opportunity to potentially give access to valuable treatments when the alternatives are scarce. In both cases, abemaciclib, a CDK4/6-inhibitor, was administered based on preclinical and phase I evidence supporting *CDKN2B* deletions as a potential biomarker of efficacy to CDK4/6 pharmacological inhibition.^41-43^

Overall, we strongly believe that the implementation of molecular tumor boards (MTB) for the correct interpretation of genomic testing results and their correct implementation in clinical practice, can optimize on-label and off-label target therapy prescriptions, as well as possible recruitment in clinical trials.^44^ In fact, MTB have already been introduced in many institutions, resulting in a significant optimization of target therapy prescriptions, as highlighted in several reports, especially for rare and/or complex tumor mutational profiles.^45-50^ Particularly interesting, in this perspective, is also the molecular tumor board portal initiative of the Cancer Core Europe consortium, based on a unified legal, scientific, and technological platform to share and harness NGS data.^46^ The ultimate goal is to automate the interpretation and reporting of complex molecular testing results, adopt a consistent expert-agreed process to systematically link tumor molecular profiles with clinical actions and reduce the need for time-consuming manual procedures, potentially prone to errors.^46^ Also, very promising seems to be the implementation of artificial intelligence-based learning programs to uniform treatment recommendations among different MTB, or directly provide treatment recommendations.^51^ Furthermore,research is however needed to deliver more solid evidence in this regard.

In our study, a high percentage of tumor samples harbored mutations involved in DNA repair mechanisms including *TP53*, *BRCA1/2*, and *ATM*. Many targeted agents directed toward these pathways are currently investigated in clinical trials or have already entered frontline clinical practice such as PARP inhibitors.^4^ In some cases, like PARP inhibitors in BC, approval has been granted in case of germline mutant alterations.^4^ Approximately 5%-10% of all solid tumors are hereditary and germline pathogenetic variants of these genes are responsible for many of currently known cancer hereditary syndromes.^52^ Although somatic genomic testing should not be performed with this purpose, our data suggest that for specific cancers and in selected cases where the oncologic family history is not available, unclear, or not particularly suspicious or when appropriate genetic counseling cannot be offered, genomic profiling might also help to identify carriers of germline cancer-associated variants. This is important, considering the implications on treatment for the patient and on cancer prevention for family members.^53^ However, suspected inheritable alterations should be further investigated with appropriate testing.

Our study has shown that a wide number of structural abnormalities are associated with genomic instability. The majority of molecular alterations identified are considered as nucleotide instabilities (NIN), a type of DNA alteration characterized by an increased frequency of substitutions, deletions, and insertions of one or few nucleotides.^54^ Following NIN, chromosomal instability (CIN) was the second most prevalent form of genomic instability identified. These findings are in line with previous studies.^55,56^ Since CIN is correlated to intrinsic multidrug resistance and poor prognosis, its detection per se might be clinically relevant and represent an additional information for the personalization of cancer care.^57,58^

It is still unclear how often genomic profiling should be repeated during the disease course because of potential genomic variability between primary and metastatic tumor, as well as between different metastatic sites.^59^ For example, within the AURORA molecular screening program an extensive profiling of BC-paired primary tumors and metastatic samples was carried out, showing an overall increase in clonality in metastatic samples.^60^ Nevertheless, when Van de Haar et al evaluated the differences in the actionable genomic landscape between biopsy pairs longitudinally collected over the treatment course in patients with different metastatic cancers,^61^ a full concordance in standard-of-care genomic biomarkers and similar ESCAT tier II mutations’ rate between the first and second biopsy was observed in 99% of the pairs.^61^ These results suggest that, while differences might be likely observed between the primary and the metastatic disease in terms of mutational profile, for the majority of metastatic cancer patients, there is a limited evolution of the actionable genome over time. Thus, a single NGS-based analysis on a metastatic sample might be both sufficient and effective to guide treatments or to evaluate a clinical trial enrollment, avoiding the need to perform multiple biopsies over time. At the same time, tumor biopsies proceed from a specific tumor tissue region and do not encompass the mutational profile of all tumor subclones, due to possible heterogeneity, not mentioning the possibility of differences across different metastatic lesions.^62^ Also, the emergence of specific subclonal genomic alterations developed under therapeutic pressure in metastatic disease could be missed, like *ESR1* mutations in HR + BC treated with aromatase inhibitors in 1st line.^63^ In this regard, liquid biopsy could offer a valuable alternative, especially when proper targeted therapeutic opportunities can be offered.^64,65^ Nevertheless, liquid biopsy is not exempt from limitations, including struggles in reliably detecting fusion and copy number events and false negatives due to low circulating tumor DNA (ctDNA) shedding, or false positives due to clonal hematopoiesis,^66,67^ as well as currently high costs.

Finally, PD-L1 is now used as a major predictive biomarker of response to ICI.^9^ Here we aimed to evaluate its association with genomic alterations in solid tumors to speculate on a potential role beyond immunotherapy. However, we did not identify any linkages between PD-L1 expression and actionable genomic alterations in our study cohort.

The main limitations of this study relate to the fact that this was performed in a single center setting, accompanied by the lack of data regarding mutation-matched treatments and patients survival outcomes, which prevented us from comprehensively assessing the practical impact of genomic profiling.

In summary, this study highlights the potential clinical value of molecular profiling in metastatic solid tumors using NGS-based panels. We observed a high overall detection rate of genomic modifications, with clinically actionable molecular alterations found in a significant proportion of cases and especially high in BC, prostate cancer, melanoma, and NSCLC. Hence, implementing molecular testing can aid in selecting patients for targeted therapies, improve treatment algorithms in situations of uncertainty, and facilitate clinical trial recruitment.

## Supplementary Material

oyae206_suppl_Supplementary_Material

## Data Availability

Anonymized data are available upon reasonable request from the corresponding authors.
