# Supplementary material for: Next-generation sequencing-based evaluation of the actionable landscape of genomic alterations in solid tumors: the “MOZART” prospective observational study
Source: Oncologist. 2024 Aug 23;30(1):oyae206. doi: 10.1093/oncolo/oyae206 (PMC11783315; doi:10.1093/oncolo/oyae206)
Supplement: oyae206_suppl_Supplementary_Material [file oyae206_suppl_supplementary_material.docx]

**Supplementary materials**

[**Supplementary references** 2](#_Toc169552119)

[**Supplementary methods** 4](#_Toc169552120)

[*Inclusion/exclusion criteria* 4](#_Toc169552121)

[*Tumor samples' specifics and requirements* 4](#_Toc169552122)

[*DNA and RNA extraction, library preparation and sequencing* 5](#_Toc169552123)

[*Quality control metrics* 5](#_Toc169552124)

[*Data analysis methods* 5](#_Toc169552125)

[*Genes covered by the assay* 6](#_Toc169552126)

[*Immunohistochemistry for PD-L1 expression - expanded* 8](#_Toc169552127)

[**Supplementary tables** 10](#_Toc169552128)

[Supplementary table 1. Somatic mutations identified 10](#_Toc169552129)

# **Supplementary references**

References adopted to confer an ESCAT scale value to each molecular alteration found in the MOZART study:

1. Mirallas, O. *et al.* New ESMO scale for clinical actionability of molecular targets (ESCAT) for gliomas based on a multicentric real world data cohort using next-generation sequencing (NGS). *JCO* 41, 2055–2055 (2023).

2. Mateo, J. *et al.* A framework to rank genomic alterations as targets for cancer precision medicine: the ESMO Scale for Clinical Actionability of molecular Targets (ESCAT). *Ann Oncol* 29, 1895–1902 (2018).

3. Condorelli, R. *et al.* Genomic alterations in breast cancer: level of evidence for actionability according to ESMO Scale for Clinical Actionability of molecular Targets (ESCAT). *Ann Oncol* 30, 365–373 (2019).

4. Verret, B., Bottosso, M., Hervais, S. & Pistilli, B. The Molecular Predictive and Prognostic Biomarkers in Metastatic Breast Cancer: The Contribution of Molecular Profiling. *Cancers (Basel)* 14, 4203 (2022).

5. Romano, P. M. *et al.* 1930O Genomic alterations in solid tumours according to ESMO scale for clinical actionability of molecular targets (ESCAT). *Annals of Oncology* 31, S1092–S1093 (2020).

6. Obermannová, R. *et al.* Oesophageal cancer: ESMO Clinical Practice Guideline for diagnosis, treatment and follow-up. *Ann Oncol* 33, 992–1004 (2022).

7. Lordick, F. *et al.* Gastric cancer: ESMO Clinical Practice Guideline for diagnosis, treatment and follow-up. *Ann Oncol* 33, 1005–1020 (2022).

8. Filetti, S. *et al.* ESMO Clinical Practice Guideline update on the use of systemic therapy in advanced thyroid cancer. *Ann Oncol* 33, 674–684 (2022).

9. Gennari, A. *et al.* ESMO Clinical Practice Guideline for the diagnosis, staging and treatment of patients with metastatic breast cancer†. *Annals of Oncology* doi:10.1016/j.annonc.2021.09.019.

10. Moreira, A. *et al.* Efficacy of molecularly targeted agents given in the randomised trial SHIVA01 according to the ESMO Scale for Clinical Actionability of molecular Targets. *Eur J Cancer* 121, 202–209 (2019).

11. Mosele, F. *et al.* Recommendations for the use of next-generation sequencing (NGS) for patients with metastatic cancers: a report from the ESMO Precision Medicine Working Group. *Ann Oncol* 31, 1491–1505 (2020).

12. ESMO/European Sarcoma Network Working Group. Gastrointestinal stromal tumours: ESMO Clinical Practice Guidelines for diagnosis, treatment and follow-up. *Ann Oncol* 25 Suppl 3, iii21-26 (2014).

13. Tamborero, D. *et al.* The Molecular Tumor Board Portal supports clinical decisions and automated reporting for precision oncology. *Nat Cancer* 3, 251–261 (2022).

14. Mulet Margalef, N. *et al.* Genomically matched therapy in refractory colorectal cancer according to ESMO Scale for Clinical Actionability of Molecular Targets: experience of a comprehensive cancer centre network. *Mol Oncol* (2023) doi:10.1002/1878-0261.13444.

15. Gan, H. K., Cvrljevic, A. N. & Johns, T. G. The epidermal growth factor receptor variant III (EGFRvIII): where wild things are altered. *FEBS J* 280, 5350–5370 (2013).

16. Tung, N. M. *et al.* TBCRC 048: Phase II Study of Olaparib for Metastatic Breast Cancer and Mutations in Homologous Recombination-Related Genes. *J Clin Oncol* 38, 4274–4282 (2020).

17. Huang, C.-C. *et al.* Comprehensive molecular profiling of Taiwanese breast cancers revealed potential therapeutic targets: prevalence of actionable mutations among 380 targeted sequencing analyses. *BMC Cancer* 21, 199 (2021).

18. Makino, E. *et al.* Targeting Rad51 as a strategy for the treatment of melanoma cells resistant to MAPK pathway inhibition. *Cell Death Dis* 11, 581 (2020).

19. Wolff, L. & Kiesewetter, B. Applicability of ESMO-MCBS and ESCAT for molecular tumor boards. *memo* 15, 190–195 (2022).

20. Andre, F. *et al.* Genomics to select treatment for patients with metastatic breast cancer. *Nature* 610, 343–348 (2022).

21. van Herpen, C. *et al.* Salivary gland cancer: ESMO-European Reference Network on Rare Adult Solid Cancers (EURACAN) Clinical Practice Guideline for diagnosis, treatment and follow-up. *ESMO Open* 7, 100602 (2022).

22. Hendriks, L. E. *et al.* Oncogene-addicted metastatic non-small-cell lung cancer: ESMO Clinical Practice Guideline for diagnosis, treatment and follow-up. *Ann Oncol* 34, 339–357 (2023).

23. Vogel, A. *et al.* Biliary tract cancer: ESMO Clinical Practice Guideline for diagnosis, treatment and follow-up. *Ann Oncol* 34, 127–140 (2023).

24. Cervantes, A. *et al.* Metastatic colorectal cancer: ESMO Clinical Practice Guideline for diagnosis, treatment and follow-up. *Ann Oncol* 34, 10–32 (2023).

# **Supplementary methods**

## *Inclusion/exclusion criteria*

For the purpose of the present analysis, the following inclusion criteria applied:

1. Female or male ≥ 18 years with diagnosis of advanced solid tumor not amenable to treatment with curative intent, after the standard second-line.
2. Written informed consent prior to registration into the molecular screening program.
3. Eastern Cooperative Oncology Group (ECOG) Performance Status 0 or 1.
4. Availability of primary tumor tissue or metastatic biopsy for research purposes, otherwise, patient must have a metastatic lesion accessible for biopsy and must agree with the biopsy procedure.

For the purpose of the present analysis, the following exclusion criteria applied:

1. Patients who had received prior palliative radiotherapy to the only site that is accessible to biopsy, if archived tissue was unavailable.
2. Presence of severe hematopoietic, renal, and/or hepatic dysfunction, including but not restricted to albumin < 3 g/dl.
3. Known increased risk of hemorrhage during biopsy procedure, as evaluated by the treating physician.
4. Previous or current malignancies of other histologies within the last 5 years, with the exception of *in situ* carcinoma of the cervix, and adequately treated basal cell or squamous cell carcinoma of the skin.

## *Tumor samples' specifics and requirements*

Patients had to present with at least one accessible metastatic lesion to perform a biopsy. If not, archived formalin-fixed paraffin-embedded (FFPE) tumor tissue from the latest biopsy received was used. The biopsies were performed at the most accessible metastatic site, as *per* standard Cremona Hospital’s protocols. They could be either computed tomography (CT)- or ultrasound (US)-guided, depending on the tumor type and biopsy site. The pathological diagnosis was established by the specific tumor-dedicated pathologist at the treating hospital. The pathologist identified the viable invasive tumor region by thoroughly reviewing H&E staining slides and meticulous circling the designated area. To ensure sufficient material for the assay, the sections required a tumor surface area of ≥ 4mm^2^ and tumor cellularity≥10%.

## *DNA and RNA extraction, library preparation and sequencing*

DNA and RNA was extracted from FFPE curls cut at 10μm or from 5μm sections mounted onto unstained glass slides using the RecoverAll^TM^ extraction kit (Ambion, Cat no.A26069). RNA samples were diluted to 5ng/μl and reverse transcribed to cDNA in a 96 well plate using the Superscript Vilo cDNA synthesis kit (CAT 11754250). The quantity of DNA from the extracted samples were measured using the Qubit^®^ 3.0 fluorometer and Qubit^®^ dsDNA High Sensitivity Assay kit (Cat:Q32854). 1µl of DNA combined with 199µl of combined HS buffer and reagent were used in qubit assay tubes for measurement. 10µl of standard 1 or 2 were combined with 190µl of the buffer and reagent solution for the controls. DNA samples were diluted to 5ng/µl and added to 5X Ion Ampliseq Hi-fi (from the From the Ion Ampliseq^TM^ library kit plus (4488990) , nuclease free water and set up using two DNA primer pools (5µl of pool 1 and 5µl of pool 2) in a 96 well plate. Library construction, template preparation, template enrichment and sequencing were performed using Ion Ampliseq^TM^ library 2.0 (Cat:4480441) and the Ion 540^TM^ OT2 kit (Cat: A27753) according to the manufacturers instructions. Sequencing was performed using the Ion S5 system^TM^ 20 (Cat: A27212) utilising Ion 540^TM^ chips (Cat:30 A27766).

## *Quality control metrics*

Sequencing runs were quality controlled using the following parameters: chip loading >60% with >45 million reads observed, enrichment 98-100%, polyclonal percentage <55%, low quality <26%, usable reads > 30% and aligned bases were ≥80%, unaligned bases were <20%, mean raw accuracy was >99% and overall read length between 100-115bp for DNA and RNA. Individual DNA sample metrics were evaluated using the following parameters: number of mapped reads >4.5 million, percent reads on target >90%, average base coverage depth >1200, uniformity of amplicon (base) coverage >90%, amplicons were required to have less than 90% strand bias with >80% of amplicons reading end to end, on-target reads >85% and target base coverage at 1x, 20x, 100x and 500x >90%.

## *Data analysis methods*

Sequence alignment and variant calling was performed on The Torrent Suite^TM^ Software (5.8.0). Alignment in Torrent Suite^TM^ Software was performed using TMAP. The output BAM file was uploaded via the IonReporterUploader plugin (5.8.32-1) to The Ion Reporter^TM^ Software (5.10.1.0). The results of variant annotation were organized hierarchically by gene, alteration, indication and level of evidence in relation to clinical actionability following the joint recommendation of the association of the AMP/ASCO/CAP (Li MM et al. *J Mol Diagn.* 2017; 19(1):4-23) and the ESMO Scale for Clinical Actionability of Molecular Targets including FDA/EMA approved therapies, guideline references by ESMO/NCCN and clinical trials (Phases I-IV) worldwide. For this reason, the test is dynamically updated every 12 weeks to incorporate potential changes in drug approval status and clinical trials progresses worldwide.

Hotspot variants with >10% alternate allele reads were classified as ‘detected’ with an assay sensitivity and positive predictive value (PPV) of 99%. For copy number variants (CNV), amplifications of CN> 6 with the 5% confidence value of ≥4 after normalization and deletions with 95% CI ≤1 were classified as present when the tumor content was >50% with a sensitivity of 80% and PPV of 100%. Gene fusions were reported when occurring in >40 counts and meeting the threshold of assay specific internal RNA quality control with a sensitivity of 92% and PPV of 99%.

Tumor mutational burden (TMB), defined as the number of somatic mutations per megabase (Mb) of interrogated genomic sequence, was calculated and provided along with mutational data results. Ten mutations/Mb was the cut-off to define high vs. low TMB cases.

## *Genes covered by the assay*

- Coding sequence (CDS) only genes:

*CALR, CIITA, CYP2D6, ERCC5, FAS, ID3, KLHL13, MTUS2, PSMB10, PSMB8, PSMB9, RNASEH2C, RPL22, RPL5, RUNX1T1, SDHC, SOCS1, STAT1, TMEM132D, UGT1A1, ZBTB20.*

- Hotspot genes:

*ACVR1, ATP1A1, BCR, BMP5, BTK, CACNA1D, CD79B, CSF1R, CTNNB1, CUL1, CYSLTR2, DGCR8, DROSHA, E2F1, EPAS1, FGF7, FOXL2, FOXO1, GLI1, GNA11, GNAQ, HIF1A, HIST1H2BD, HIST1H3B, HRAS, IDH1, IL6ST, IRF4, IRS4, KLF4, KNSTRN, MAP2K2, MED12, MYOD1, NSD2, NT5C2, NTRK2, NUP93, PAX5, PIK3CD, PIK3CG, PTPRD, RGS7, RHOA, RPL10, SIX1, SIX2, SNCAIP, SOS1, SOX2, SRSF2, STAT5B, TAF1, TGFBR1, TRRAP, TSHR, WAS.*

- Hotspot Genes and Copy Number Variation:

*ABL1, ABL2, AKT1, AKT2, AKT3, ALK, AR, ARAF, AURKA, AURKC, AXL, BCL2, BCL2L12, BCL6, BRAF, CARD11, CBL, CCND1, CCND2, CCND3, CCNE1, CDK4, CDK6, CHD4, DDR2, EGFR, EIF1AX, ERBB2, ERBB3, ERBB4, ESR1, EZH2, FAM135B, FGFR1, FGFR2, FGFR3, FGFR4, FLT3, FLT4, FOXA1, GATA2, GNAS, H3F3A, H3F3B, IDH2, IKBKB, IL7R, KDR, KIT, KLF5, KRAS, MAGOH, MAP2K1, MAPK1, MAX, MDM4, MECOM, MEF2B, MET, MITF, MPL, MTOR, MYC, MYCN, MYD88, NFE2L2, NRAS, NTRK1, NTRK3, PCBP1, PDGFRA, PDGFRB, PIK3C2B, PIK3CA, PIK3CB, PIK3R2, PIM1, PLCG1, PPP2R1A, PPP6C, PRKACA, PTPN11, PXDNL, RAC1,RAF1, RARA, RET, RHEB, RICTOR, RIT1, ROS1, SETBP1, SF3B1, SLCO1B3, SMC1A, SMO, SPOP, SRC, STAT3, STAT6, TERT, TOP1, TPMT, U2AF1, USP8, XPO1, ZNF217, ZNF429.*

- CNV Gain Genes:

*ABCB1, CTNND2, DDR1, EMSY, FGF19, FGF23, FGF3, FGF4, FGF9, FYN, GLI3, IGF1R, MCL1, MDM2, MYCL, RPS6KB1, RPTOR, YAP1, YES1.*

- CNV Loss and Coding sequence (CDS):

*ABRAXAS1, ACVR1B, ACVR2A, ADAMTS12, ADAMTS2, AMER1, APC, ARHGAP35, ARID1A, ARID1B, ARID2, ARID5B, ASXL1, ASXL2, ATM, ATR, ATRX, AXIN1, AXIN2, B2M, BAP1, BARD1, BCOR, BLM, BMPR2, BRCA1, BRCA2, BRIP1, CASP8, CBFB, CD274, CD276, CDC73, CDH1,  CDH10, CDK12, CDKN1A, CDKN1B, CDKN2A, CDKN2B, CDKN2C, CHEK1, CHEK2, CIC, CREBBP, CSMD3, CTCF, CTLA4, CUL3, CUL4A, CUL4B, CYLD, CYP2C9, DAXX, DDX3X, DICER1, DNMT3A, DOCK3, DPYD, DSC1, DSC3, ELF3, ENO1, EP300, EPCAM, EPHA2, ERAP1, ERAP2, ERCC2, ERCC4, ERRFI1, ETV6, FANCA, FANCC, FANCD2, FANCE, FANCF, FANCG, FANCI, FANCL, FANCM, FAT1, FBXW7, FUBP1, GATA3, GNA13, GPS2, HDAC2, HDAC9, HLA-A,  HLA-B, HNF1A, INPP4B, JAK1, JAK2, JAK3, KDM5C, KDM6A, KEAP1, KMT2A, KMT2B, KMT2C, KMT2D, LARP4B, LATS1, LATS2, MAP2K4, MAP2K7, MAP3K1, MAP3K4, MAPK8, MEN1, MGA,  MLH1, MLH3, MRE11, MSH2, MSH3, MSH6, MTAP, MUTYH, NBN, NCOR1, NF1, NF2, NOTCH1, NOTCH2, NOTCH3, NOTCH4, PALB2, PARP1, PARP2, PARP3, PARP4, PBRM1,   PDCD1, PDCD1LG2, PDIA3, PGD, PHF6, PIK3R1, PMS1, PMS2, POLD1, POLE, POT1, PPM1D,  PPP2R2A, PRDM1, PRDM9, PRKAR1A, PTCH1, PTEN, PTPRT, RAD50, RAD51, RAD51B,  RAD51C, RAD51D, RAD52, RAD54L, RASA1, RASA2, RB1, RBM10, RECQL4, RNASEH2A,   RNASEH2B, RNF43, RPA1, RUNX1, SDHA, SDHB, SDHD, SETD2, SLX4, SMAD2, SMAD4, SMARCA4, SMARCB1, SOX9, SPEN, STAG2, STK11, SUFU, TAP1, TAP2, TBX3, TCF7L2, TET2,  TGFBR2, TNFAIP3, TNFRSF14, TP53, TP63, TPP2, TSC1, TSC2, USP9X, VHL, WT1, XRCC2,  XRCC3, ZFHX3, ZMYM3, ZRSR2.*

- Gene fusions:

*AKT2, ALK, AR, AXL, BRAF, BRCA1, BRCA2, CDKN2A, EGFR, ERBB2, ERBB4, ERG, ESR1, ETV1,  ETV4, ETV5, FGFR1, FGFR2, FGFR3, FGR, FLT3, JAK2, KRAS, MDM4, MET, MYB, MYBL1, NF1, NOTCH1, NOTCH4, NRG1, NTRK1, NTRK2, NTRK3, NUTM1, PDGFRA, PDGFRB, PIK3CA, PPARG, PRKACA, PRKACB, PTEN, RAD51B, RAF1, RB1, RELA, RET, ROS1, RSPO2, RSPO3, TERT.*

- TMB-only genes:

*A1CF, ACSM2B, ADAM18, ANO4, ARMC4, BRINP3, C6, C8A, C8B, CANX, CASR, CD163,CNTN6, CNTNAP4, CNTNAP5, COL11A1, DCAF4L2, DCDC1, GALNT17, GPR158, GRID2,  HCN1, HLA-C, KCND2, KCNH7, KEL, KIR3DL1, KRTAP2-1, KRTAP6-2, LRRC7, MARCO, NLRC5, NOL4, NRXN1, NYAP2, OR10G8, OR2G6, OR2L13, OR2L2, OR2L8, OR2M3, OR2T3,   OR2T33, OR2T4, OR2W3, OR4A15, OR4C15, OR4C6, OR4M1, OR4M2, OR5D18, OR5F1,   OR5L1, OR5L2, OR6F1, OR8H2, OR8I2, OR8U1, ORC4, PAK5, PCDH17, PDE1A, PDE1C, PLXDC2, POM121L12, PPFIA2, RBP3, REG1A, REG1B, REG3A, REG3G, RPTN, RUNDC3B,  SH3RF2, SLC15A2, SLC8A1, SYT10, SYT16, TAPBP, TPTE, TRHDE, TRIM48, TRIM51, ZIM3, ZNF479, ZNF536.*

## *Immunohistochemistry for PD-L1 expression - expanded*

The propietary Immunofocus PD-L1 IHC assay was centrally applied to quantify the proportion of tumor cells that express PD-L1 (tumor proportion score) and the area occupied by tumor infiltrating PD-L1 positive immune cells in FFPE samples. The assay is a Laboratory Developed Test utilizing the RUO rabbit monoclonal antibody clone E1L3N (Cell Signalling Technologies) and Leica Bond III instrumentation. The assessment of PD-L1 immunostaining was performed by a qualified histopathologist at Oncologica^®^ (G.W.) in accordance with PD-L1 clinical reporting guidelines^13^. According to the tumor proportional score (TPS), all cases were subdivided in PD-L1 negative (TPS<1%), positive-low (TPS ≤1%≤TPS≤49%) and positive-high (TPS ≥50%)^14,15^. The performance of the Immunofocus assay is continually assessed by involvement in recognized External Quality Assessment schemes and returns performance levels commensurate with the approved PD-L1 diagnostic assays. However, the clone E1L3N is not licensed and approved for use in clinical testing to direct the use of PD1/PD-L1 therapies.

# **Supplementary tables**

## **Supplementary table 1. Somatic mutations identified**

| Gene | Mutation | | Gene | Mutation | | Gene | Mutation | |  |
| --- | --- | --- | --- | --- | --- | --- | --- | --- | --- |
| *AKT1* | p.(E17K) | c.49G>A | *FANCI* | p.(G401fs) | c.1202delG | *PIK3CA* | p.(R93Q) | c.278G>A |  |
| *AKT1* | p.(L52R) | c.155T>G | *FBXW7* | mutation |  | *PIK3CA* | p.(R93W) | c.277C>T |  |
| *AKT2* | amplification |  | *FBXW7* | p.(R224*) | c.670C>T | *PIK3CA* | p.(T1025S) | c.3073A>T |  |
| *AKT3* | amplification |  | *FBXW7* | p.(R465C) | c.1393C>T | *PIK3R1* | mutation |  |  |
| *AR* | amplification |  | *FBXW7* | p.(R465H) | c.1394G>A | *PTCH1* | mutation |  |  |
| *ARID1A* | deletion |  | *FGF* | aberration |  | *PTEN* | deletion |  |  |
| *ARID1A* | mutation |  | *FGF19* | amplification |  | *PTEN* | p.(C296fs) | c.886delT |  |
| *ARID1A* | p.(D972fs) | c.2914delG | *FGF3* | amplification |  | *PTEN* | p.(G127*) | c.379G>T |  |
| *ARID1A* | p.(E1227*) | c.3679G>T | *FGFR1* | amplification |  | *PTEN* | p.(H196fs) | c.586_593delCACAAGAT |  |
| *ARID1A* | p.(E2246*) | c.6736G>T | *FGFR1* | fusion |  | *PTEN* | p.(K6*) | c.16A>T |  |
| *ARID1A* | p.(E2250fs) | c.6748_6752delGAGTT | *FGFR1* | p.(N577K) | c.1731C>A | *PTEN* | p.(Q245*) | c.733C>T |  |
| *ARID1A* | p.(Q581*) | c.1741C>T | *FGFR2* | p.(Y375C) | c.1124A>G | *PTEN* | p.(Q87*) | c.259C>T |  |
| *ARID1A* | p.(R110fs) | c.329_332delGGCC | *FGFR3* | p.(F384L) | c.1150T>C | *PTEN* | p.(Y88*) | c.264T>G, |  |
| *ARID1A* | p.(S735fs) | c.2203_2204insA | *FGFR3* | p.(R248C) | c.742C>T | *PTEN* | p.(A126S) | c.376G>T |  |
| *ATM* | deletion |  | *FGFR3* | p.(S249C) | c.746C>G | *PTPN11* | mutation |  |  |
| *ATM* | mutation |  | *FGFR3-TACC3* | fusion |  | *PTPN11* | p.(A72T) | c.214G>A |  |
| *ATM* | p.(E1892*) | c.5674G>T | *FGFR4* | amplification |  | *RAD50* | deletion |  |  |
| *ATM* | p.(F763fs) | c.2289delT | *FLT3* | amplification |  | *RAD50* | mutation |  |  |
| *ATM* | p.(R2993*) | c.8977C>T | *GNAS* | p.(R201H) | c.602G>A | *RAD50* | p.(Y625*) | c.1875C>G |  |
| *ATM* | p.(R805*) | c.2413C>T | *IDH1* | p.(R132H) | c.395G>A | *RAD51B* | mutation |  |  |
| *ATM* | p.(V1268*) | c.3802delG | *KDR* | amplification |  | *RAD51C* | mutation |  |  |
| *ATM* | p.(V128*) | c.381delA | *KDR* | mutation |  | *RAD51D* | p.(F97fs) | c.290delT |  |
| *ATR* | deletion |  | *KIT* | amplification |  | *RB1* | deletion |  |  |
| *ATR* | mutation |  | *KIT* | mutation |  | *RB1* | p.(E275fs) | c.823_824insG |  |
| *ATR* | p.(E560*) | c.1678G>T | *KRAS* | A146 mutation |  | *RB1* | p.(S501fs) | c.1503_1512delTACATCTCAG | |
| *ATRX* | p.(E649*) | c.1945G>T | *KRAS* | G12 mutation |  | *RNF43* | p.(R145*) | c.433C>T | |
| *AXL* | amplification |  | *KRAS* | G12D mutation |  | *SETD2* | deletion |  | |
| *BAP1* | deletion |  | *KRAS* | G12V mutation |  | *SETD2* | mutation |  | |
| *BAP1* | mutation |  | *KRAS* | G13 mutation |  | *SMARCB1* | p.(E360*) | c.1078G>T | |
| *BAP1* | p.(Q694*) | c.2079_2080delGCinsAT | *KRAS* | amplification |  | *SMO* | mutation |  | |
| *BAP1* | p.(T69fs) | c.206delC | *KRAS* | exon 2 mutation |  | *STK11* | deletion |  | |
| *BRAF* | V600E mutation | mutation | *KRAS* | p.(A146T) | c.436G>A | *STK11* | mutation |  | |
| *BRAF* | amplification |  | *KRAS* | p.(G12A) | c.35G>C | *STK11* | p.(E120*) | c.358G>T | |
| *BRAF* | p.(K601E) | c.1801A>G | *KRAS* | p.(G12C) | c.34G>T | *TBL1XR1-PIK3CA* | fusion |  | |
| *BRAF* | p.(V600E) | c.1799T>A | *KRAS* | p.(G12D) | c.35G>A | *TMEM178B-BRAF* | fusion |  | |
| *BRAF* | p.(V600K) | c.1798_1799delGTinsAA | *KRAS* | p.(G12R) | c.34G>C | *TP53* | exon 8 mutation |  | |
| *BRCA1* | mutation |  | *KRAS* | p.(G12S) | c.34G>A | *TP53* | mutation |  | |
| *BRCA1* | p.(?) | c.547+2T>A | *KRAS* | p.(G12V) | c.35G>T | *TP53* | p.(A86fs) | c.255_277delTGCACCAGCCCCCTCCTGGCCCC | |
| *BRCA1* | p.(L502fs) | c.1504_1507delTTAA | *KRAS* | p.(G13D) | c.38G>A | *TP53* | p.(C124fs) | c.371_372insG | |
| *BRCA2* | deletion |  | *KRAS* | p.(Q61H) | c.183A>C | *TP53* | p.(C141Y) | c.422G>A | |
| *BRCA2* | mutation |  | *KRAS* | p.(Q61H) | c.183A>T | *TP53* | p.(C176F) | c.527G>T | |
| *BRCA2* | p.(G267fs) | c.800delG | *MDM2* | amplification |  | *TP53* | p.(C238Y) | c.713G>A | |
| *BRCA2* | p.(H1932fs) | c.5796_5797delTA | *MET* | amplification |  | *TP53* | p.(C242F) | c.725G>T | |
| *BRCA2* | p.(K3326*) | c.9976A>T | *MET* | exon 14 skipping |  | *TP53* | p.(C275W) | c.825T>G | |
| *BRCA2* | p.(N1377_T1378ins*) | c.4131_4132insTGAGGA | *MET* | fusion |  | *TP53* | p.(D281V) | c.842A>T | |
| *BRCA2* | p.(Q2561fs) | c.7680_7681insT | *MET* | mutation |  | *TP53* | p.(E204*) | c.610G>T | |
| *BRCA2* | p.(R2394*) | c.7180A>T | *MET* | p.(R988C) | c.2962C>T | *TP53* | p.(E271*) | c.811G>T | |
| *BRCA2* | p.(R2659*) | c.7975A>T | *MET* | p.(T1010I) | c.3029C>T | *TP53* | p.(E285K) | c.853G>A | |
| *BRCA2* | p.(S1982fs) | c.5946delT | *MSH6* | p.(W413*) | c.1238G>A | *TP53* | p.(E326*) | c.976G>T | |
| *BRCA2* | p.(S401*) | c.1202C>G | *MTOR* | mutation |  | *TP53* | p.(E346*) | c.1036G>T | |
| *BRCA2* | p.(Y1894*) | c.5682C>G | *MTOR* | p.(E1799K) | c.5395G>A | *TP53* | p.(G112fs) | c.335delG | |
| *CAPZA2-MET* | fusion |  | *MTOR* | p.(S2215F) | c.6644C>T | *TP53* | p.(G244V) | c.731G>T | |
| *CBL* | p.(R420Q) | c.1259G>A | *MTOR* | p.(S2215Y) | c.6644C>A | *TP53* | p.(G245D) | c.734G>A | |
| *CCDC6-RET* | fusion |  | *MYC* | amplification |  | *TP53* | p.(G245S) | c.733G>A | |
| *CCND1* | amplification |  | *MYD88* | mutation |  | *TP53* | p.(G266R) | c.796G>A | |
| *CCND1* | mutation |  | *NBN* | mutation |  | *TP53* | p.(H179fs) | c.535_538delCATG | |
| *CCND2* | amplification |  | *NBN* | deletion |  | *TP53* | p.(K132R) | c.395A>G | |
| *CCNE1* | amplification |  | *NF1* | mutation |  | *TP53* | p.(L265fs) | c.793_812delCTGGGACGGAACAGCTTTGA | |
| *CDK12* | p.(M1173fs) | c.3518_3525delTGGCCCCA | *NF1* | p.(E1423*) | c.4267G>T | *TP53* | p.(M237I) | c.711G>A | |
| *CDK4* | amplification |  | *NF1* | p.(M799_E800delinsI*) | c.2397_2398delGGinsTT | *TP53* | p.(N200fs) | c.599delA | |
| *CDK6* | amplification |  | *NF1* | p.(R2083fs) | c.6248delG | *TP53* | p.(N268fs) | c.801delG | |
| *CDKN1B* | p.(E53fs) | c.157delG | *NF1* | p.(S1355*) | c.4064C>G | *TP53* | p.(P278L) | c.833C>T | |
| *CDKN2A* | deletion |  | *NF1* | p.(S2719*) | c.8156C>G | *TP53* | p.(P58fs) | c.173delC | |
| *CDKN2A* | mutation |  | *NF1* | p.(W696*) | c.2087G>A | *TP53* | p.(Q317fs) | c.950_963delAGCCAAAGAAGAAA | |
| *CDKN2A* | p.(H83Y) | c.247C>T | *NF1* | p.(Y2285fs) | c.6852_6855delTTAC | *TP53* | p.(R156fs) | c.466delC | |
| *CDKN2B* | deletion |  | *NF1* | p.(Y408*) | c.1224T>A | *TP53* | p.(R175G) | c.523C>G | |
| *CHEK2* | mutation |  | *NF2* | p.(Q115*) | c.343C>T | *TP53* | p.(R175H) | c.524G>A | |
| *CREBBP* | mutation |  | *NF2* | p.(Q178*) | c.532C>T | *TP53* | p.(R196*) | c.586C>T | |
| *EGFR* | A289V mutation | mutation | *NFE2L2* | mutation |  | *TP53* | p.(R248G) | c.742C>G | |
| *EGFR* | amplification |  | *NOTCH* | aberration |  | *TP53* | p.(R248Q) | c.743G>A | |
| *EGFR* | fusion |  | *NOTCH* | mutation |  | *TP53* | p.(R248W) | c.742C>T | |
| *EGFR* | mutation |  | *NTRK1* | amplification |  | *TP53* | p.(R249G) | c.745A>G | |
| *EGFR* | p.(A289V) | c.866C>T | *NTRK3* | fusion |  | *TP53* | p.(R273H) | c.818G>A | |
| *EGFR* | p.(G719D) | c.2156G>A | *PDGFRA* | amplification |  | *TP53* | p.(R280G) | c.838A>G | |
| *EGFR* | vIII |  | *PDGFRA* | fusion |  | *TP53* | p.(R280I) | c.839G>T | |
| *EGFR-SEPT14* | fusion |  | *PDGFRB* | amplification |  | *TP53* | p.(R280T) | c.839G>C | |
| *ERBB2* | amplification |  | *PIK3CA* | H1047R mutation |  | *TP53* | p.(R282W) | c.844C>T | |
| *ERBB2* | p.(G776delinsVC) | c.2326_2327insTCT | *PIK3CA* | N345 mutation |  | *TP53* | p.(R306*) | c.916C>T | |
| *ERBB2* | p.(L755S) | c.2264T>C | *PIK3CA* | aberration |  | *TP53* | p.(S241F) | c.722C>T | |
| *ERBB2* | p.(S310F) | c.929C>T | *PIK3CA* | fusion |  | *TP53* | p.(S314fs) | c.940delT | |
| *ERBB3* | p.(A232V) | c.695C>T | *PIK3CA* | mutation |  | *TP53* | p.(V173L) | c.517G>T | |
| *ERBB3* | p.(E928G) | c.2783A>G | *PIK3CA* | p.(E542K) | c.1624G>A | *TP53* | p.(V173M) | c.517G>A | |
| *ERBB3* | p.(M60K) | c.179T>A | *PIK3CA* | p.(E545G) | c.1634A>G | *TP53* | p.(V216E) | c.647T>A | |
| *ERCC2* | mutation |  | *PIK3CA* | p.(E545K) | c.1633G>A | *TP53* | p.(V216M) | c.646G>A | |
| *ESR1* | mutation |  | *PIK3CA* | p.(H1047L) | c.3140A>T | *TP53* | p.(V272M) | c.814G>A | |
| *ESR1* | p.(D538G) | c.1613A>G | *PIK3CA* | p.(H1047R) | c.3140A>G | *TP53* | p.(Y220C) | c.659A>G | |
| *ESR1* | p.(V392I) | c.1174G>A | *PIK3CA* | p.(M1043V) | c.3127A>G | *TP53* | p.(Y234H) | c.700T>C | |
| *ESR1* | p.(Y537S) | c.1610A>C | *PIK3CA* | p.(N345H) | c.1033A>C | *TP53* | p.(Y236H) | c.706T>C | |
| *ESR1* | p.(Y537S) | c.1610A>C | *PIK3CA* | p.(N345K) | c.1035T>A | *TSC1* | deletion |  | |
| *FANC* | deletion |  | *PIK3CA* | p.(Q546K) | c.1636C>A | *TSC2* | deletion |  | |
| *FANCD2* | deletion |  | *PIK3CA* | p.(Q546R) | c.1637A>G | *WHSC1L1-FGFR1* | fusion |  | |
